# Phylogenetics of the Fascial System

**DOI:** 10.7759/cureus.10787

**Published:** 2020-10-04

**Authors:** Leonardo Vieira

**Affiliations:** 1 Osteopathy, Brazilian Academy of Fascias, Belo Horizonte, BRA

**Keywords:** phylogenetic, fascial system, osteopathic medicine, fascia, physiotherapy

## Abstract

The fascial system, due to its enormous capacity to connect all other body systems, is currently highlighted for a better understanding of human life and health. The evolutionary theory is the most accepted explanation today to describe the development of this enormous variety of life on our planet. The report presents phylogenesis through the eyes of the fascial system. The development of the fascial system and its adaptations have made it possible to increase Homo sapiens' survival and success. We present a historical contextualization of the evolutionary theory followed by the main changes in the movement fasciae, in the transverse diaphragms, visceral fasciae, dermis, subcutaneous tissue, and neural fasciae. The article presents the evolutionary perspective with the resulting increase in efficiency with less energy expenditure.

## Introduction and background

Charles Darwin, along with Alfred Wallace, proposed that all individuals came from a single ancestor. In his book, On the Origin of Species, published in 1858, Darwin impacted society at the time by presenting the theory of natural selection [1]. With the advancement of technology, some principles of this theory have undergone changes. The genetic revolution has also brought in new concepts and information about the evolutionary theory.

However, due to his importance in the field of evolutionary science, Darwin is considered the “father of evolution.” According to Douglas Theobald, in a work published in 2010, the probability of all living beings having a single common ancestor is 102,860 times greater than the possibility of biological diversity having presented independent paths [2]. Hence, the evolutionary hypothesis has an enormous weight in the understanding of how we, Homo sapiens, have become the most adapted species on planet Earth.

Natural selection is a process composed of three common phenomena:

1) Principle of variation: every organism differs from others of the same species.

2) Genetic heredity: some variations present in all populations are inherited, transmitted genetically from parents to their children.

3) Differential reproductive success: all organisms, including humans, differ in the number of descendants they produce, which survive to reproduce.

Genetic modifications in individuals of a species that generate survival advantages increase reproductive chances and, consequently, generate more adapted descendants. The evolutionary process is stimulated mainly in times of scarcity. These advantages transmitted to the descendants and added to other acquisitions over many generations are passed on to an increasing group of individuals who, at any given moment, are no longer able to reproduce with the individuals of the old population. At this moment, a new species appears [3].

Basic precepts for life will always be taken into consideration to guide our discussion: the lower the energy expenditure, the greater the chance of survival (less allostatic load).

To make this possible, several adaptive events have occurred in all systems throughout the evolution of species. Few published works have explored the fascial system from an evolutionary perspective. The aim of this paper is to understand the importance of this system in the interaction with all the systems of the body and, at the same time, forming a link between all structures. The current definition of the fascial system as per the Fascia Research Society is as follows: “the fascial system consists of the three-dimensional continuum of soft, collagen-containing, loose and dense fibrous connective tissues that permeate the body. It incorporates elements such as adipose tissue, adventitia and neurovascular sheaths, aponeuroses, deep and superficial fasciae, epineurium, joint capsules, ligaments, membranes, meninges, myofascial expansions, periostea, retinacula, septa, tendons, visceral fasciae, and all the intramuscular and intermuscular connective tissues including endo-/peri-/epimysium. The fascial system surrounds, interweaves between, and interpenetrates all organs, muscles, bones, and nerve fibers, endowing the body with a functional structure, and providing an environment that enables all body systems to operate in an integrated manner” [4].

Knowledge about the phylogenesis of the fascial system would allow for a more productive assessment and intervention in promoting the individual’s health. Although indivisible, we will use a didactic organizational segmentation used in several publications. We will divide this into four major topics: fasciae of movement, visceral fasciae, superficial fasciae, and neural fasciae.

## Review

Historical context

A major evolutionary milestone for sapiens was the use of the bipedal position for locomotion [5]. There is strong evidence to show that this advance was initiated at a time of major changes on our planet. Estimates indicate that between five and eight million years ago, there was a major climate change that affected the Earth. In East Africa, a forest-rich region, a great period of cooling changed the availability of natural resources. Until that fact, a great abundance of fruits and food had been available in those forests. This climatic change modified the entire local environmental structure, including the reduction of natural food resources for the great diversity of species that inhabited this region. There was high mortality of several species. Driven by the survival instinct, several species of animals left their natural environment to seek resources in other regions. Several of these species left in search of another location that would provide a greater chance of survival. Some species of animals reached the African savanna. Our emphasis will be given to the Homo genus to seek relations with our evolution.

In a more scarce environment, energy expenditure for locomotion in search of food becomes an important factor in natural selection. Some individuals of the Homo genus started to move in a bipedal position, of course not with the same skill as our current one. Measurements of energy expenditure show that bipedal stance actually consumes about four times less energy than quadrupedal stance in individuals with the same body weight [6]. Individuals who were able to travel long distances using less energy possibly obtained more food and increased their chance of survival. From several generations influenced by natural selection, a new species of individuals originated, our cousins ​​in common with the chimpanzees, the Australopithecus, the first to use the bipedal position most of the time [7]. However, they presented great difficulties such as very flat feet, short legs, and rib cage shape still very similar to that of the chimpanzee. The gait still had a lot of lateral inclination, demanding a high energy expenditure compared to the human gait, but already much smaller than that of a chimpanzee [6]. These events bring a historical understanding to comprehend our phylogenetic adaptations. Chimpanzees are our closest living “cousins”, with whom we share more than 98% of our genetic code [8]. From this point, we will generate a comparison in relation to the movement fasciae in order to understand our advantages.

Movement fasciae

**Figure 1:**
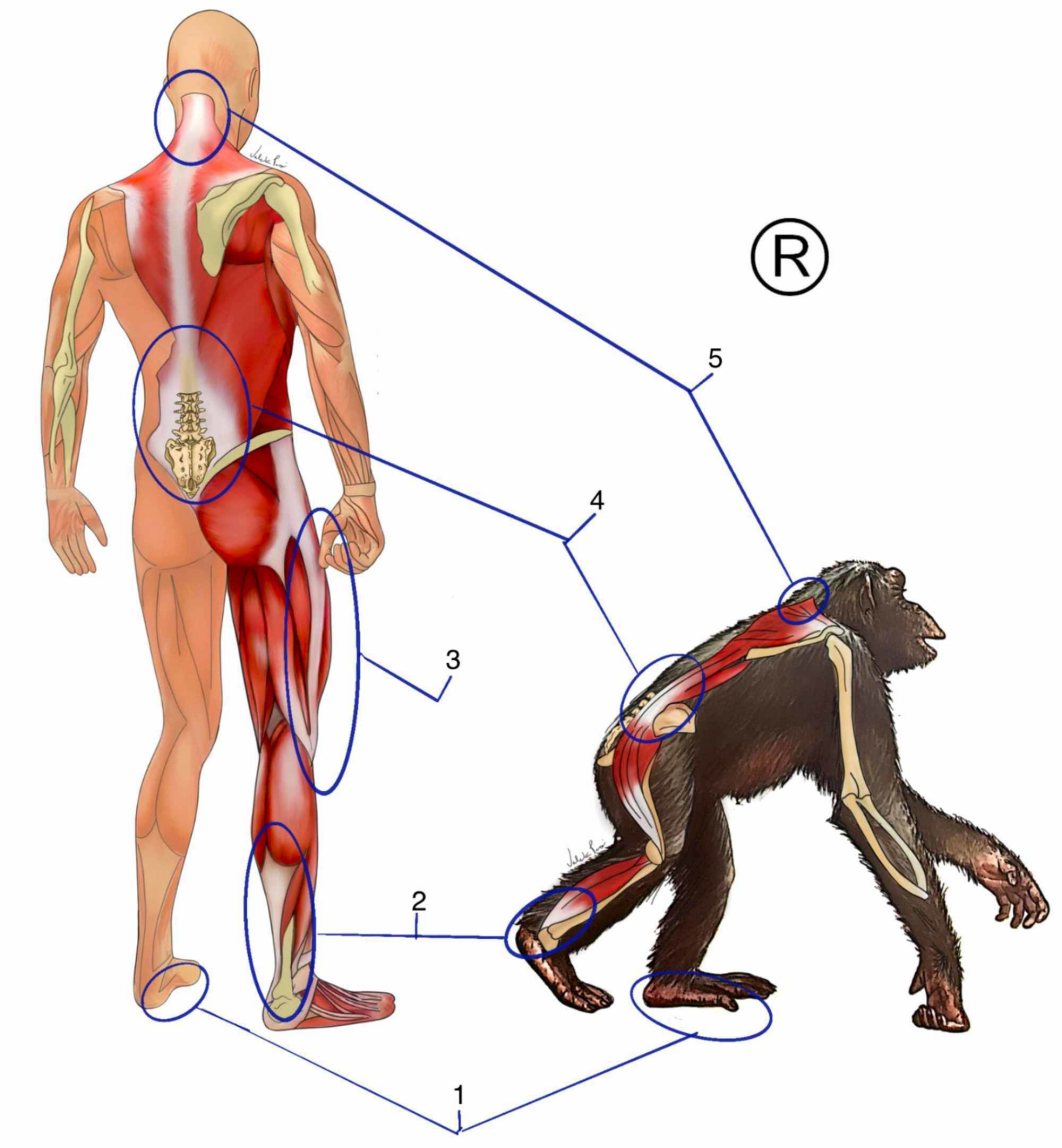
Phylogenetics of movement fasciae 1: plantar fascia; 2: Achilles tendon; 3: iliotibial band; 4: thoracolumbar fascia; 5 nuchal ligament The figure belongs to the author's database. Copyright reserved by the author

Several musculoskeletal changes were necessary to adapt to the bipedal position. Skeletal changes such as changes in the shape of the bones of the foot, femur with enlarged valgus, enlargement of the pelvis, presence of a waist, and the appearance of another lumbar vertebra (Figure 1). The organization of the spine in a curvature system with an inverted compensatory pattern was fundamental for changing the gravitational axis in the bipedal position [9].

We present a plantar arch associated with an extremely efficient plantar fascia for transmitting force in relation to chimpanzees. When we walk, run, or jump, the plantar fascia takes advantage of the reaction force of the soil and transmits it upwards through the Achilles tendons [10]. Comparing the size of the human Achilles tendon with that of the chimpanzee, we observed a much larger tendon (Figure 1). It is formed by the fascial expansions of the soleus, gastrocnemius, and plantar muscles [11]. In humans, the plantar muscle becomes less functional, and in some cases, it is not present at all. Also called the triceps surae tendon, it presents continuity with the posterior layer of the crural fascia [12]. Upon reaching the knee, a sophisticated center of fascial interaction, formed by retinacula, tendons, and ligaments, it transmits tension and modulates the support system, especially in the single-legged phase of gait.

An incredible variation of synovial bursae that interconnect, enabling great efficiency in protecting the joint and propagating the kinetic energy that ignites through the entire fascia lata, was formed by all the fasciae of the thigh [12]. The lateral part of the thigh is formed by a fibrous septum, the iliotibial tract, seen only in Homo sapiens, which leaves the lateral part of the tibia through the femur, where it is continuous with the fasciae of the gluteus maximus, gluteus medius, and tensor fascia lata (Figure 1). The iliotibial tract combined in its positional relationship with the gluteus fascia allowed to reduce the lateral trunk inclination in the single-legged support [13]. The gluteal fascia superiorly inserts itself into the thoracolumbar fascia, continuing the path of force transmission. The thoracolumbar fascia allows spine stabilization, control of the center of gravity, and synchronism between the pelvic and scapular girdle [14]. The thoracolumbar fascia is formed by the meeting of the fasciae of the abdominal muscles with those of the hip with the posterior torso. Its organization in the human species is so complex that we do not have a uniform anatomical classification. We will adopt the two-layer model mentioned by Willard et al. in 2012 [15].

We have an anterior and a posterior layer. The posterior part is subdivided into two compartments from the transverse process of the lumbar vertebrae: the most superficial layer of the posterior part, which is formed by the fascia of the gluteus maximus with the fascia of the reverse latissimus dorsi, acting on the control of movement mainly in the frontal and transverse planes. The deepest part of the posterior layer involves the erector muscles of the spine and the segmental muscles. In the lower region, it presents insertion in the periosteum of the sacrum, continuity with the sacrotuberous ligament, hamstring tendon, and fascia latae. Superiorly, it is continuous until the base of the skull. This region of the thoracolumbar fascia mainly controls movements in the sagittal plane, such as flexion-extension. In front of the transverse process, we have the anterior layer of the thoracolumbar fascia. This layer involves the psoas and quadratus lumborum muscles. Its adaptations allow an excellent ratio of the increased movements in the pelvis and trunk, especially those of extension. At the same time, this layer has an important relationship with the retroperitoneal organs, mainly the genitourinary system and the renal fascia [16]. Following the movement, the fascia of the trapezius muscle is continuous with the fascia of the latissimus dorsi, inserting itself superiorly into the entire nuchal ligament and also in the occipital bone [12] (Figure 1). The nuchal ligament is a fascial junction of all the posterior fascial layers of the trunk linking all the cervical spine processes and the first two thoracic vertebrae [17]. This ligament interferes positively in the control of shear forces and in the formation of cervical lordosis generated by the new body alignment. In the upper cervical region, this ligament is continuous with the dura mater in the base of the skull [18]. Through this mechanism and all of the aforementioned chain, we can assume that the single-legged support can function as a way of pumping by alternating tension in the region of the skull base through the connection of the nuchal ligament with the dura mater in that region. This mechanism possibly favors the maintenance of venous flow through the jugular foramen, keeping intracranial pressure stable during exhausting activities.

Bipedal stance allowed us to free our hands and upper limbs. This was important for us to develop great movements of the scapular girdle and limbs. The upper limb fasciae have a unique organization, allowing for a wide range and diversity of movements [13]. The upper limb is adapted to work in open kinetic chain movements, different from the reality presented by quadrupeds. As a consequence, the making of tools and the control of fire increased the chance of survival [3]. The complexity and efficiency of the tools increased as the skills and physical changes took place. This was accompanied by a great neurofascial organization giving an increasingly precise refinement to the distal movements of the upper limb [19]. The wrist left the quadrupedal flexion position for a wide variety of movements, allowing adjustments for fine movements [3]. An organization of the palmar fasciae into five independent layers associated with a large concentration of proprioceptive receptors in the retinacula on the wrist gives the sapiens precision [20].

The release of the scapular girdle for hunting movements, as a food uptake resource, was essential to increase the range of motion of the entire upper limb [21]. This gain in the release of the scapular waist, in addition to the refined coordination promoted by the thoracolumbar fascia, allowed great efficiency in handling the spear. This object allowed hunting at a distance, compensating for the sapiens’ decreased speed and, at the same time, decreasing the risk of corporal combat with prey, often infinitely stronger [3]. During a race, we have a great efficiency of this fascial system in the use of energy. The ankle retinacules are integrated in order to adjust to this demand, presenting a higher concentration of proprioceptors when compared to quadruped primates. In addition, the compartments of the feet assume a different configuration, giving up a gripping function, mainly by the hallux, for movements of extension and transmission of force in order to propel the body forward as a locomotion strategy [22].

Studies with kangaroos have shown a fundamental contribution of the fascial system to the efficiency of the locomotion movement [23]. When studying humans, the same power transmission capacity and kinetic energy savings demonstrated in gait, jump and run studies were proven. Kawakami et al., in 2002, demonstrated that at the moment of running in the deformation of the fascial system, the passive component was much larger than the musculoskeletal system, the active component. Even with great articular amplitudes, the movement occurred with the muscles contracting almost in an isometric way, while the fascial system presented great deformations, transmitting the force for the subsequent movements. This generates great energy savings through the mechanical and neuromuscular pathways [10].

In an analysis of the center of gravity, when jumping on unstable surfaces, it was demonstrated that a large part of the correction came from the passive component, generating great savings by decreasing the use of neuromuscular strategies [24]. The iliotibial tract stores about 7 joules of elastic energy per stride while running, saving energy for subsequent movements [25]. This also influences the strategy of maintaining the body without much oscillation in the single-legged support, since the corrections of inclinations of the body consume energy. The execution of eye movements in all directions and maintaining complete independence of the head and neck is an optimized feature in sapiens [26]. In addition to adaptations in the fascial system, through the Tenon capsule, there was an advanced neuromuscular control associated with changes in the eye structure itself [13-27].

The diaphragms

Our species is the only one that has five connective tissue structures anatomically arranged in a transversal form forming the five diaphragms of the body. They are, from cranial to caudal, as follows: the tentorium cerebelli, tongue (floor of the mouth), upper thoracic diaphragm, muscular diaphragm, and pelvic diaphragm (pelvis floor) [28]. These structures have a mechanical and neural connection, enabling several important functions for the bipedal position. They form limits of interdependent body cavities that, at the same time, need autonomy to perform various individual functions at different times. The five diaphragms provide us with control and synchronization of the intracavitary pressures, acting on the fluidic circulation between the cavities and also in the interstitium of the visceral parenchyma [28].

The tongue and floor of the mouth are innervated by five cranial pairs that direct their information to nuclei of the brain stem [29]. Mechanically it has continuity with all the fascias of the digestive tract and respiratory tract, reaching the base of the skull where it becomes continuous with the dura mater [30]. The upper thoracic diaphragm is formed by the upper portion of the endothoracic fascia and the junction of the parietal pleura with the deep layer of the deep fascia. It is innervated by the sympathetic spinal nerves. The new positional relationships of the clavicle, scapula, and shoulder promoted major adaptations. Only in sapiens does the junction between a movement fascia and a visceral fascia occur, forming the endothoracic fascia [31].

The diaphragm muscle in the quadrupeds helps in the movement of the torso laterality in locomotion. In sapiens, it plays an important role in synchronizing with the pelvic diaphragm, controlling the movements of the lower limb through the connection with the psoas muscle, in the new position, through the medial arcuate ligament [32]. The lumbar part helps to stabilize the lumbar region, especially in the second half of the forced expiration [33]. In quadrupeds, the diaphragm has a limited relationship with the pericardium and the heart. In bipeds, it starts to assume an important role in support, with greater influence on cardiac performance. It also plays an important role in tensioning the pharynx and larynx, participating in events such as speech and vomiting [34]. The action of reflux control by the diaphragm is enhanced in the bipedal position. The pelvic diaphragm is formed by the muscles and fasciae: ilium, ischium, and pubococcygeus, together with the piriformis fascia and obturator fascia being innervated by the sacral nerves [12]. Brainstem nuclei coordinate constant reflexes for synchronism, seeking to orchestrate the optimization of the body's functioning [28]. In the cranial fasciae session, we will detail the cerebellum tent more.

Visceral fasciae

**Figure 2:**
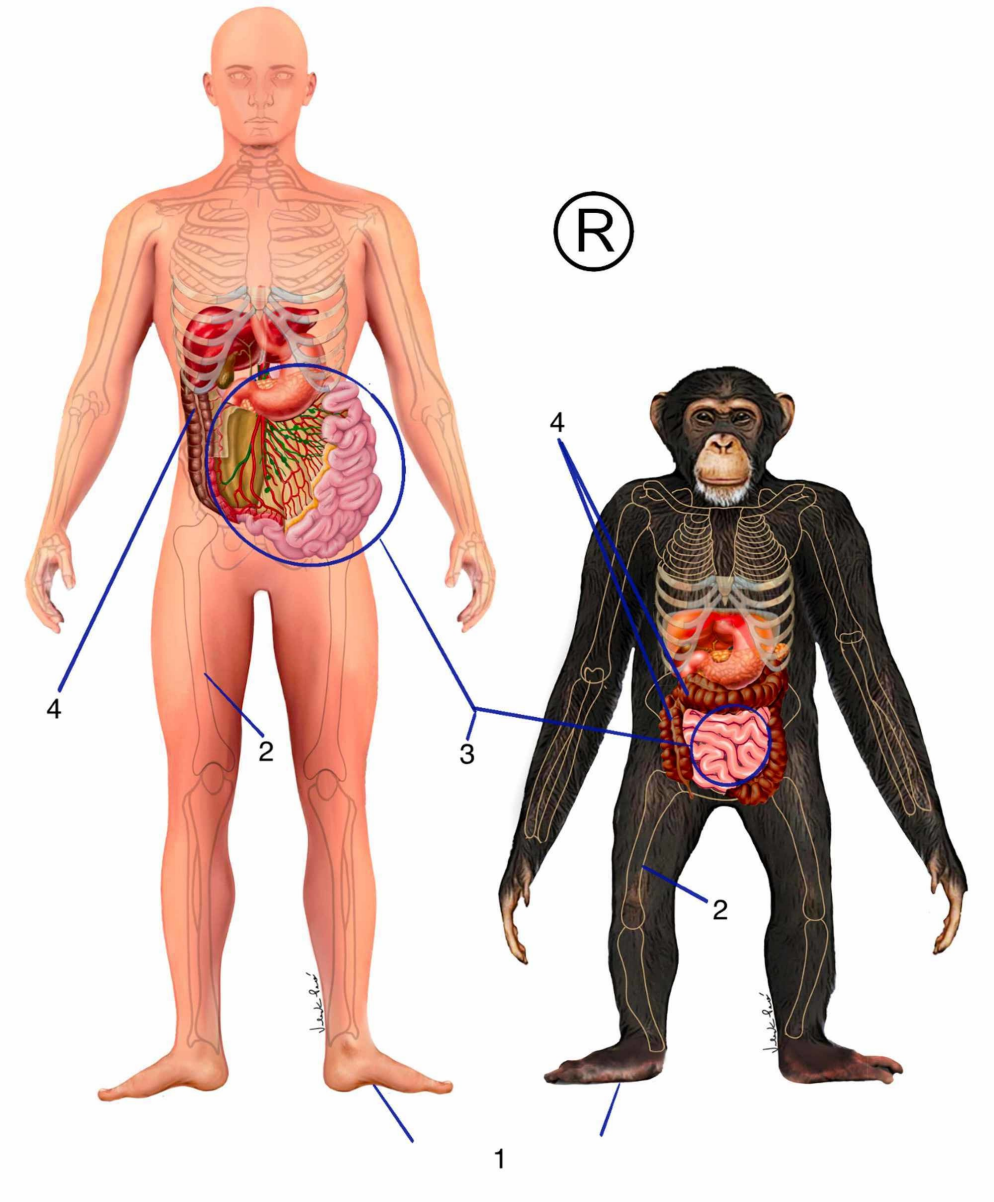
Phylogenetics of visceral fasciae 1: plantar fascia; 2: femur position; 3: small intestine and mesentery fascia; 4: large intestine The figure belongs to the author's database. Copyright reserved by the author

The two systems that consume the most energy in the body are the digestive and the cerebral [3]. The primitive digestive system expends a lot of energy to break down food and make it usable for the body. Using strategies such as fermentation, lower mammals and even primates have organs such as voluminous large intestine (Figure 2). A visceral fascial organization is automatically adapted to the new digestive demands [31].

The effects of our ancestors’ experience on the African savanna environment resulted in major visceral changes. With food shortages, a great diversity of new foods was incorporated into the diet. The roots became a potential food with a lot of important nutrients [21]. At the same time, an increase in masticatory muscle mass and, automatically, a thickening of the respective fasciae of these muscles came as a consequence of the new demand. The connective tissue that forms the musculoskeletal structures on the face of mammals develops from the growth of the digestive system. The development of foregut releases gene factors responsible for attracting neural crest cells to form the connective tissue part of that region [35]. In response to the demand, individuals genetically stronger in their chewing presented an evolutionary advantage. This automatically reflects in a protrusion of the maxilla and mandible and consequently a retraction of the frontal part of the skull. As manual skills evolve, more powerful tools are manufactured [3].

The bipedal position changed the entire positioning relationship of the spine and viscera. With the horizontalization of the skull base, there is a change in the insertion axis of the buccopharyngeal fascia, changing the positioning and functioning of the nasopharynx, pharynx, hyoid bone, and larynx [26-30]. This alters the functional relationships in speech production, alters the spatial relationships for the development of the tongue and the hyoid, both in the digestive function and in the articulation of several different types of sounds. The relationship between tongue and epiglottis in sapiens is different from all other mammals. The space between the base of the tongue and the soft palate is greater in sapiens [26].

We have a lower and shorter larynx compared to other primates. Our babies have a high, nasal larynx, like most adult mammals, favoring gas exchange in the first months of life. In addition, human babies have a limited degree of neuromuscular control. The intracranial larynx decreases the risk of aspirating milk or saliva. By the third month, the larynx is already in a lower position. The rapid growth of the mandibular and maxillary arches together with the development of the skull and cervical base, associated with the development of the digestive and respiratory systems, results in a repositioning of the larynx, located below the hyoid [26]. The middle layer of the deep cervical fascia together with the fasciae of the mandible, the floor of the mouth and tongue are remodeled to the new positioning, and also to the new functions. A smaller tongue, however, as well as movements and a very efficient sensory and muscular system increased the variety of possibilities. The tongue is continuous with visceral fasciae that follow the digestive tract inferiorly and also superiorly to the base of the skull [30]. In this region, the connective tissue continues with the dura mater that surrounds the foramen of the passage of the vessels that penetrate the skull [36]. It also has continuity with the visceral fasciae of the respiratory tube and larynx, providing extremely diverse sound production. The angle between the nasopharynx and the respiratory tube is close to 90 degrees and with the acquisition of the external nose turned downwards, associated with a peculiar arrangement of the nasal conchae of the internal nose, creating a favorable environment for humidification and heating of the air, and enabling greater efficiency in gas exchange at the pulmonary level. At the same time, due to this disposition, during great efforts, where the breathing becomes more intense, there is a considerable increase in the flow and this starts to have a turbulent trajectory. This allows a much larger amount of air to have more contact with the surface of the nasal mucosa and consequently a greater concentration of air that reaches the alveoli with the potential to be captured [26].

At the cervical level, we have a new organization of the middle layer of the deep fascia, also known as pretracheal fascia. This fascia is formed by two laminae: the first and most superficial involves the infrahyoid muscles and is important for the new mechanisms of language and the deglutition relationship of the bipedal posture. The deepest lamina surrounds the thyroid and the parathyroid presenting continuity with the pericardium, forming the suspensory ligaments of the pericardium [36]. This structure is important due to gravity. In addition, the thyroid has undergone important developments to adjust to the new metabolic demand and its role is fundamental in the circadian rhythm. Its hormones act on metabolism and also on the pineal gland influencing tissue replacement in the body [37].

The bipedal position has important consequences for the development of visceral fasciae. A very important phylogenetic adaptation in sapiens is the change in the relationship between the parietal lung pleura and the fasciae of the trunk. In lower mammals, when moving around at a speed, they use the respiratory strategy always linked to the positioning of limbs and thorax. In the high-speed phase when they reach trunk extension and maximum limb distance, the inspiratory moment occurs, and the expiratory moment occurs at the moment of trunk flexion with the approach of the limbs [3]. Despite the efficiency from the speed point of view, this respiratory link to musculoskeletal movements prevents the strategy of gasp, which constitutes an efficient internal cooling mechanism. When moving at high speed for a long time, the internal organs overheat, causing the system to collapse. In sapiens, the parietal pleura merges with the deep intercostal fascia. This allows the independence of the respiratory moment with the movement of the trunk and limbs. At the same time that the sapiens lose speed jogging, the ability to run for longer distances increases. This all adds up to refrigeration strategies promoted by changes in the superficial fasciae and dermis, as we will see in the respective section of this article. The independence of breathing in relation to the movement system and phonation allows greater efficiency in all of these systems.

Bipedalism brought about refinement to the movements of the upper limbs. As a result of this came the dominance of fire and the improvement of hunting tools, bringing significant changes in hunting and in the conditions for preparing food. Large animals, protein, and animal fat brought important energy subsidies to new demands. Owing to this, our ancestors started to use the masticatory apparatus much less, because the food became much softer [3]. This caused a decrease in the size of the muscles and fasciae of the masticatory apparatus. A consequent retraction of the viscerocranium, maxilla, and mandible gave space for the frontal region, the neurocranium, to evolve. Digestive strategies, such as fermentation, became less important in the body. The large intestine decreased its size and there was an increase in the size of the small intestine and consequently a larger area for nutrient absorption (Figure 2) [38]. The increased food diversity also generated an increase in the number of microorganisms establishing a symbiotic relationship in the small intestine, because, in addition to helping with digestion, they released several important substances that acted in the development of the brain [39]. With all this, the energy balance underwent major changes. Less energy was spent on digestion, more nutrients were absorbed by the system. The frontal region now had a greater possibility of growth and available energy for this to occur [3].

Important changes have also occurred in the pelvic visceral fasciae. The action of gravity in the bipedal position and the increase in the width of the pelvis changed visceral relations, and especially of the organs and fasciae of the smaller pelvis. The increased lateral diameter minimized the pressure effect of the bipedal position [3]. In addition, changes occurred in the suspensory ligaments of pelvic organs. Substantial changes occurred at the level of the pelvic diaphragm in order to sustain the imposed demand. At that moment, a dissipation of force by the intravisceral movements between the insertional fasciae, which constitutes each outermost visceral fascial, was able to minimize gravitational effects. For this, sapiens had an increase in loose connective tissue and intravisceral fat. An increase in the size of the small intestine associated with a bipedal position generated important changes in the mesentery. The visceral fascia that surrounds the jejunum and ileum was adjusted to adapt to a considerable increase in the vascular network for a greater intake of nutrients [31-38].

Dermis and subcutaneous tissue

The dermis and subcutaneous tissue are part of a large fascial system that involves the entire body [4]. Major acquisitions in the dermis and subcutaneous tissue have become fundamental to the adaptive success of sapiens [40].

The dermis is the layer located just below the epidermis, participating in the constitution of the skin. It represents the most innervated fascial tissue in the human body. It presents high concentrations of primitive, polymodal neural receptors, very simple in their physical constitution [41]. These receptors are called interceptors that control the conditions of the body’s internal environment, informing the central nervous system of various types of information including nociceptive [42]. The great evolution of sapiens is the destination of this information. The information travels from the skin to the posterior horn of the spinal cord, making the first synapse ascending to the upper centers, with the final destination being the insular cortex. The soft dermal touch promotes a feeling of pleasure and well-being in the mammals related to conviviality and interaction. These interactions are even stronger in Homo sapiens. Human beings have reached the peak of social interactions, greatly increasing the chance of survival. This type of sensation promoted by light touch is also called sensual touch and also promotes the stimulation of higher centers linked to sexuality [43]. These two acquisitions meet the two laws of the evolutionary principle: survival and reproduction. They also have a large concentration of phylogenetically more evolved receptors, encapsulated, and with the presence of fat in their composition, such as Meissner, Merkel, Pacini, and Ruffini [44]. This was very important in the proprioceptive and exteroceptive refinement, managing to improve the positioning relationships of the body in space and the perceptions of the external environment.

In the sapiens dermis, there is an average of 5-10 million sweat glands spread throughout the body and which constitute an efficient cooling system, which we will explain below [3]. Primates have sweat glands in specific areas of the body such as the feet, hands, and genitals. The subcutaneous tissue is basically composed of two layers of fat separated by a layer of loose connective tissue, the superficial fascia itself. The most superficial layer of fat establishes a direct relationship with the dermis. It presents an organized collagen network arranged perpendicularly to the skin, with the optimized function in the matter of impact cushioning. The deepest layer of fat has a direct relationship with the deep layer of the deep fascia, which is the first layer of movement. It presents an organization of collagen with an oblique disposition favoring the sliding between the layers, mitigating the effects of muscle contraction and movement fasciae on the most superficial layers [12].

The subcutaneous tissue layer, in addition to being richly innervated, has a vast vascular network. About 70% of the body’s lymphatic and venous network is in this tissue [31]. The interaction between this tissue and the dermis promotes a great survival advantage for sapiens in relation to all other animals. In exhaustive physical activities or in very hot climatic conditions, such as in African savannas, a large amount of sweat produced by the dermal sweat glands is spread throughout the body, including the head region. When sweat comes into contact with air, it cools the skin. This has a cooling effect on the large blood circulation in the superficial veins of the subcutaneous tissue. This blood flows throughout the body, cooling the organs and also the brain. This mechanism is essential for maintaining extreme conditions, preventing the internal system from collapsing due to overheating [26]. This advantage was fundamental to our hunter-gathering ancestors. Bipedal running is less efficient in terms of speed than sprinting. However, when moving at speed, the quadrupeds are unable to use their main cooling mechanism, the gasping. As a result, our ancestors chased prey until their system collapsed due to overheating. Hence they were easily slaughtered [21]. In the cerebral part, there were other adaptations that we will highlight in the section on the evolution of the cranial fasciae.

Sapiens presents a mimic musculature with a great diversity of movements, an important evolutionary development for social organization. In the face region, we have a junction of the superficial fascia with the movement fascia [12].

Neural fasciae

The advent of the bipedal position brought about several changes in the fascial system of the human head and cervical spine, maintaining the horizontality of the look and, at the same time, adequate stability of the skull and cervical joint. The occipital condyles are designed to allow a very little movement. Strong ligament complexes are incorporated into the region [26]. Important relationships, already present in primates, have been refined, such as, for example, the connection with the dura mater at the base of the skull. The fascia of the occipital muscles and the nuchal ligament have direct insertions in the dura mater in the upper cervical [45]. In the frontal region of the skull, the epicranial fascia interconnects the base of the skull with the Tenon capsule, in the orbital region. The Tenon capsule is continuous with the cranial dura mater, allowing direct mechanical coordination between the base and the eye region [27]. These connections facilitate the synchronism and, at the same time, the independence of the movements of the head with the movements of the eyeball. In addition, sapiens present a high concentration of proprioceptive receptors in the muscular fasciae, mainly in the perimysium of the suboccipital muscles [46]. This neurofascial interaction allows for a refined synchronization of skull control during postural demands and movement.

**Figure 3:**
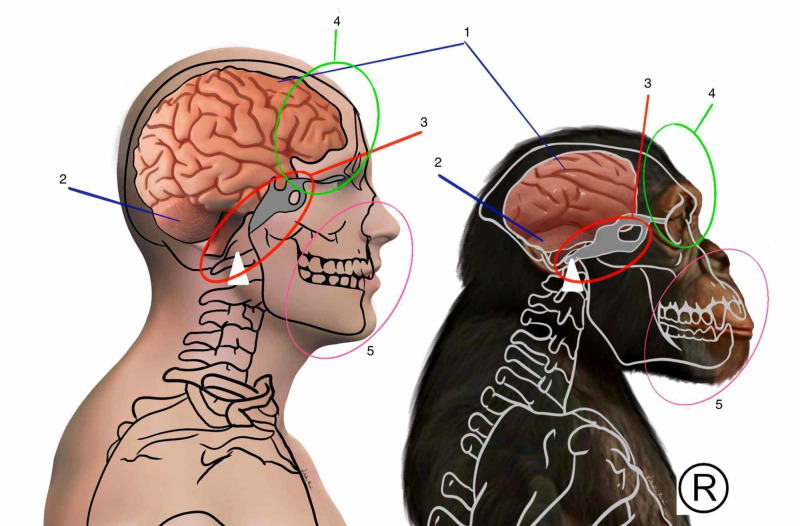
Comparison between the human cortex and the chimpanzee cortex 1: cortex; 2: cerebellum; 3: relationship of sphenoid with foramen magnum; 4: frontal cortex; 5: maxilla and mandible Copyright for the image reserved by the author

Studies of head development analysis demonstrate that the development of the anterior part of the cortex, especially the frontal cortex, is stimulated by gene factors released from the development of the digestive tract [35]. It is likely that the evolution of sapiens’ foregut in the bipedal position directly influenced the great cortical evolution of our species. This demonstrates the difficulty of segmenting human systems because, in reality, the development of one structure directly or indirectly influences the development of another, even though they belong to different systems. The cerebral cortex shows an enormous growth in the sapiens, practically doubling in relation to the chimpanzee (Figure 3). There was a great growth of the frontal and prefrontal cortex, with the acquisition of nuclei, which allowed the capacity for deep conscious analysis, a great capacity for abstraction, future projections, and increasingly organized social interactions. Broca's and Wernicke's areas present a large increase in volume in relation to primates allowing a great evolution of the language and communication system in general [26-37].

In the anterior region of the skull, the retraction of the visceral cranium was accompanied by a protrusion of the frontal region. This promoted a change in the angles of the sphenobasilar synchondrosis and in the angle between the orbit and the cranial base (Figure 3). Consequently, the organization of the dural compartments has also undergone changes [26]. The tension lines of the falx of the brain, formed by the upper part of the dura mater altered its conformation due to the change in the position of the ethmoid and consequently the crista galli, where the falx is inserted. The falx of the brain subsequently converges to the internal occipital protuberance. In mammals, this region has a more posterior location in relation to primates due to the greater development of cortical, cerebellar areas, and changes in the development of the squamous part of the occipital bone (Figure 3) [47]. The region of the internal occipital protuberance also receives the insertion of the cerebellum falx, which divides and organizes the cerebellum into two hemispheres at a horizontal level. At the horizontal level, the change in the organization of the sphenoid promotes a change in the diaphragm of the sella turcica, which represents one of the parts formed by the invagination of the meningeal dura mater, where the pituitary is located. The tentorium cerebelli represents the fourth dural organization [34]. It presents a horizontal arrangement fixing laterally on the petrous crests of the temporal, previously on the lower wings of the sphenoid, more precisely in the anterior clinoid processes. It follows posteriorly around the edge of the posterior cranial fossa bilaterally, meeting in the internal occipital protuberance, at the border between the endochondral part and the intramembranous part of the occipital bone. Its structure is greatly modified in sapiens to support the large increase in cortical weight in conjunction with the change in the center of gravity. Part of this support is given by the other parts of the dura mater that present continuity, distributing the stresses to all pillars through the organization of collagen fibers forming dural bands of force transmission [18].

The dura mater actively participates in regulating the ossification of the bones at the apex of the head. The region of the sagittal and coronal sutures is where the greatest growth occurs in sapiens. Dura mater acts as a mediator in the relationship between very high brain growth and the rapid development of the bones of the cranial vault [47]. This dural adaptation allowed greater efficiency in the shape and function of the venous sinuses. The confluence of the sinuses became more superior, together with the straight sinus, increasing the efficiency of the brain’s cranial drainage system [48].

At the peripheral level, with the increase in the possibility of movement, the mechanical interfaces between the peripheral nerves and the tissues to which they pass have also undergone modifications. The mesoneurium, made up of loose connective tissue, is reinforced in places of greater mobility. A greater number of receptors and more specific ones have been incorporated, mainly in the joint tissue, to organize increasingly complex movements and tension lines. At the medullary level, increasingly sophisticated modulation processes were generating several adjustments in the conjunctive system without the participation of the cortex [19].

At the brainstem level, synchronized control between body systems has been incorporated in addition to the possibility of isolated actions of various systems, greatly increasing the possibility of functioning by peripheral control [19]. At this level, modulatory reactions have been improved, preventing various information from being processed by the brain, generating great energy savings. A great evolution in neuronal nuclei such as the ambiguous nucleus allowed a greater possibility of facial expressions, together with the control of cervical movements and refinement in pressure control. This nucleus coordinates information regarding the nerves: vagus, spinal accessory, glossopharyngeal, and facial [49]. The cerebellum holds about 80% of the brain’s neuronal bodies, refining control and motor coordination, helping to refine existing movements at the same time and allowing the execution of increasingly complex movements [50].

The ligaments between the medulla and the medullary canal were remodeled in relation to the new demand. The curvatures of the spine modified the relations of the medulla and meninges with the medullary canal. Adaptations in the anterior ligaments, such as the yellow and anterior longitudinal, were necessary in order to allow an increase in mobility of the spine, especially in the extension movement. At the cranial level, major changes occurred allowing adaptations for cortical growth and organization of brain areas. Although the region of the base of the skull is considered to be older from a phylogenetic point of view, the horizontalization of the foramen magnum greatly changed the meningeal relations of the region, providing an organization that promotes stability and functionality in the high cervical transition and brain bulb. The occipital bone is formed by mesodermal cells from the first four somites, called occipital somites [26].

## Conclusions

The fascial system, due to its function of interconnecting all the systems of the body, has great importance in the mechanism of operation and energy saving. Understanding the phylogenetic evolution of this system allows us to expand the possibilities of identifying dysfunctional zones responsible for the increase in energy expenditure leading to an increase in the allostatic load and, consequently, assuming pathologies. The evolutionary knowledge of the fascial system allows us to design conducts in order to optimize the characteristics that make human beings the most adapted species on the planet. We hope that this study will provide a stimulus to researchers to seek further clarifications regarding the evolutionary processes of the fascial system, especially given the scarcity of publications on the subject.

## References

[1] Darwin C (2020). On the Origin of Species: The Science Classic. Edited by John van Wyhe. Capstone, A Wiley Brand, Padstow, UK.

[2] Theobald DL (2010). A formal test of the theory of universal common ancestry. Nature.

[3] Lieberman DE (2013). The Story of the Human Body: Evolution, Health, and Disease. https://books.google.co.in/books/about/The_Story_of_the_Human_Body.html?id=Ht_zugAACAAJ&redir_esc=y.

[4] Schleip R, Hedley G, Yucesoy CA (2019). Fascial nomenclature: update on related consensus process. Clin Anat.

[5] Zachos J, Pagani M, Sloan L, Thomas E, Billups K (2001). Trends, rhythms, and aberrations in global climate 65 Ma to present. Science.

[6] Sockol MD, Raichlen DA, Pontzer H (2007). Chimpanzee locomotor energetics and the origin of human bipedalism. Proc Natl Acad Sci U S A.

[7] DeSilva JM, Holt KG, Churchill SE, Carlson KJ, Walker CS, Zipfel B, Berger LR (2013). The lower limb and mechanics of walking in Australopithecus sediba. Science.

[8] Prüfer K, Munch K, Hellmann I (2012). The bonobo genome compared with the chimpanzee and human genomes. Nature.

[9] Bramble DM, Lieberman DE (2004). Endurance running and the evolution of Homo. Nature.

[10] Kawakami Y, Muraoka T, Ito S, Kanehisa H, Fukunaga T (2002). In vivo muscle fibre behaviour during counter-movement exercise in humans reveals a significant role for tendon elasticity. J Physiol.

[11] Huijing PA, Jaspers RT (2005). Adaptation of muscle size and myofascial force transmission: a review and some new experimental results. Scand J Med Sci Sports.

[12] Stecco C (2015). Functional Atlas of the Human Fascial System. https://books.google.co.in/books/about/Functional_Atlas_of_the_Human_Fascial_Sy.html?id=8eDTBQAAQBAJ&source=kp_book_description&redir_esc=y.

[13] Eng CM, Arnold AS, Biewener AA, Lieberman DE (2015). The human iliotibial band is specialized for elastic energy storage compared with the chimp fascia lata. J Exp Biol.

[14] Barker PJ, Hapuarachchi KS, Ross JA, Sambaiew E, Ranger TA, Briggs CA (2014). Anatomy and biomechanics of gluteus maximus and the thoracolumbar fascia at the sacroiliac joint. Clin Anat.

[15] Willard FH, Vleeming A, Schuenke MD, Danneels L, Schleip R (2012). The thoracolumbar fascia: anatomy, function and clinical considerations. J Anat.

[16] Tozzi P, Bongiorno D, Vitturini C (2012). Low back pain and kidney mobility: local osteopathic fascial manipulation decreases pain perception and improves renal mobility. J Bodyw Mov Ther.

[17] (2016). Gray's Anatomy: The Anatomical Basis of Clinical Practice, 41st Edition. https://www.elsevier.ca/ca/product.jsp?isbn=9780702052309.

[18] Humphreys BK, Kenin S, Hubbard BB, Cramer GD (2003). Investigation of connective tissue attachments to the cervical spinal dura mater. Clin Anat.

[19] Butler AB, Hodos W (2005). Comparative Vertebrate Neuroanatomy: Evolution and Adaptation.

[20] Stecco C, Macchi V, Lancerotto L, Tiengo C, Porzionato A, De Caro R (2010). Comparison of transverse carpal ligament and flexor retinaculum terminology for the wrist. J Hand Surg Am.

[21] Santurbano P (2017). Human Evolution and Movement: Introduction to Evolutionary Reasoning in Health and Movement (Book in Portuguese). Edited: Santurbano Pablo. Publishing: Autor, São Paulo, Brazil.

[22] Ker RF, Bennett MB, Bibby SR, Kester RC, Alexander RM (1987). The spring in the arch of the human foot. Nature.

[23] Kram R, Dawson TJ (1998). Energetics and biomechanics of locomotion by red kangaroos (Macropus rufus). Comp Biochem Physiol B Biochem Mol Biol.

[24] Moritz CT, Farley CT (2004). Passive dynamics change leg mechanics for an unexpected surface during human hopping. J Appl Physiol (1985).

[25] Eng CM, Arnold AS, Lieberman DE, Biewener AA (2015). The capacity of the human iliotibial band to store elastic energy during running. J Biomech.

[26] Lieberman DE (2011). The Evolution of the Human Head. https://www.hup.harvard.edu/catalog.php?isbn=9780674046368.

[27] Kakizaki H, Takahashi Y, Nakano T (2012). Anatomy of Tenons capsule. Clin Exp Ophthalmol.

[28] Bordoni B (2020). The five diaphragms in osteopathic manipulative medicine: myofascial relationships, part 1. Cureus.

[29] Bordoni B, Morabito B, Mitrano R, Simonelli M, Toccafondi A (2018). The anatomical relationships of the tongue with the body system. Cureus.

[30] Warshafsky D, Goldenberg D, Kanekar SG (2012). Imaging anatomy of deep neck spaces. Otolaryngol Clin North Am.

[31] Stecco L, Stecco C (2014). Fascial Manipulation for Internal Dysfunctions.

[32] Kocjan J, Adamek M, Gzik-Zroska B, Czyżewski D, Rydel M (2017). Network of breathing. Multifunctional role of the diaphragm: a review. Adv Respir Med.

[33] Helsmoortel J, Hirth T, Wührl P (2010). Visceral Osteopathy: Peritoneal Organs. Edition: Kaplan A, Bensky D. Eastland.

[34] Bordoni B, Simonelli M, Morabito B (2019). The fascial breath. Cureus.

[35] Le Douarin NM, Couly G, Creuzet SE (2012). The neural crest is a powerful regulator of pre-otic brain development. Dev Biol.

[36] Feigl G, Hammer GP, Litz R, Kachlik D (2020). The intercarotid or alar fascia, other cervical fascias, and their adjacent spaces - a plea for clarification of cervical fascia and spaces terminology. J Anat.

[37] (2010). Human Brain Evolution: The Influence of Freshwater and Marine Food Resources. https://www.wiley.com/en-us/Human+Brain+Evolution%3A+The+Influence+of+Freshwater+and+Marine+Food+Resources-p-9780470452684.

[38] Furness JB, Cottrell JJ, Bravo DM (2015). Comparative gut physiology symposium: comparative physiology of digestion. J Anim Sci.

[39] Sherwin E, Bordenstein SR, Quinn JL, Dinan TG, Cryan JF (2019). Microbiota and the social brain. Science.

[40] Jablonski NG (2006). Skin: A Natural History. Edited: Jablonski N.

[41] Fede C, Porzionato A, Petrelli L (2020). Fascia and soft tissues innervation in the human hip and their possible role in post-surgical pain. J Orthop Res.

[42] D'Alessandro G, Cerritelli F, Cortelli P (2016). Sensitization and interception as key neurological concepts in osteopathy and other manual medicines. Front Neurosci.

[43] McGlone F, Cerritelli F, Walker S, Esteves J (2017). The role of gentle touch in perinatal osteopathic manual therapy. Neurosci Biobehav Rev.

[44] Montagna W (1985). The evolution of human skin. J Hum Evol.

[45] Pontell ME, Scali F, Marshall E, Enix D (2013). The obliquus capitis inferior myodural bridge. Clin Anat.

[46] Liu JX, Thornell LE, Pedrosa-Domellöf F (2003). Muscle spindles in the deep muscles of the human neck: a morphological and immunocytochemical study. J Histochem Cytochem.

[47] Blechschmidt E (2020). Studies in Biodynamic Embryology. Edited: Konrad Obermeier. Kiener, Munich/ GER.

[48] Falk D (1986). Evolution of cranial blood drainage in hominids: enlarged occipital/marginal sinuses and emissary foramina. Am J Phys Anthropol.

[49] Porges SW (2007). A phylogenetic journey through the vague and ambiguous Xth cranial nerve: a commentary on contemporary heart rate variability research. Biol Psychol.

[50] Lent R (2010). One Hundred Billion Neurons: Fundamental Concepts of Neuroscience (Book in Portuguese). Edition: R. Lent. Ateneu.

